# Localization of composite prosthetic feet: manufacturing processes and production guidelines

**DOI:** 10.1038/s41598-023-44008-7

**Published:** 2023-10-13

**Authors:** Ramadan Elgamsy, Mohammed Ibrahim Awad, Noha Ramadan, Ayman Amer, Yomna Osama, Rana El-hilaly, Ahmed Elsabbagh

**Affiliations:** 1 Design and Production Engineering, Faculty of Engineering, Ain Shams University, Cairo, Egypt; 2 Mechatronics Engineering, Faculty of Engineering, Ain Shams University, Cairo, Egypt; 3 Rheumatology and Rehabilitation, Faculty of Medicine, Ain Shams University, Cairo, Egypt

**Keywords:** Mechanical engineering, Health care

## Abstract

Amputation levels in Egypt and the surrounding neighborhood require a state intervention to localize the manufacturing of prosthetic feet. Amputations are mainly due to chronic diseases, accidents, and hostilities’ casualties. The prosthetic foot type is traditionally classified according to the number of axial rotational movements, and is recently classified according to the energy activeness of the foot. The localization of this industry needs a preliminary survey of the domestic technological levels with respect to the foot type. Upon the results of this survey, the energy storage response foot has appealing metrics to proceed with its manufacturing. A prototype manufacturing chain is designed and a set of these feet with a certain commercial size of 27 is manufactured. Resin impregnation technology for carbon fiber composites is followed in this work. The feet are tested according to ISO 22,675. Based on the dimensional and mechanical results, a manufacturing value chain is proposed with the prospective resin transfer molding technology. This value chain will guarantee the required localization as well as the natural growth of this value chain with all related activities like accreditation of practices as well as manpower certification.

## Introduction

**Figure 1:**
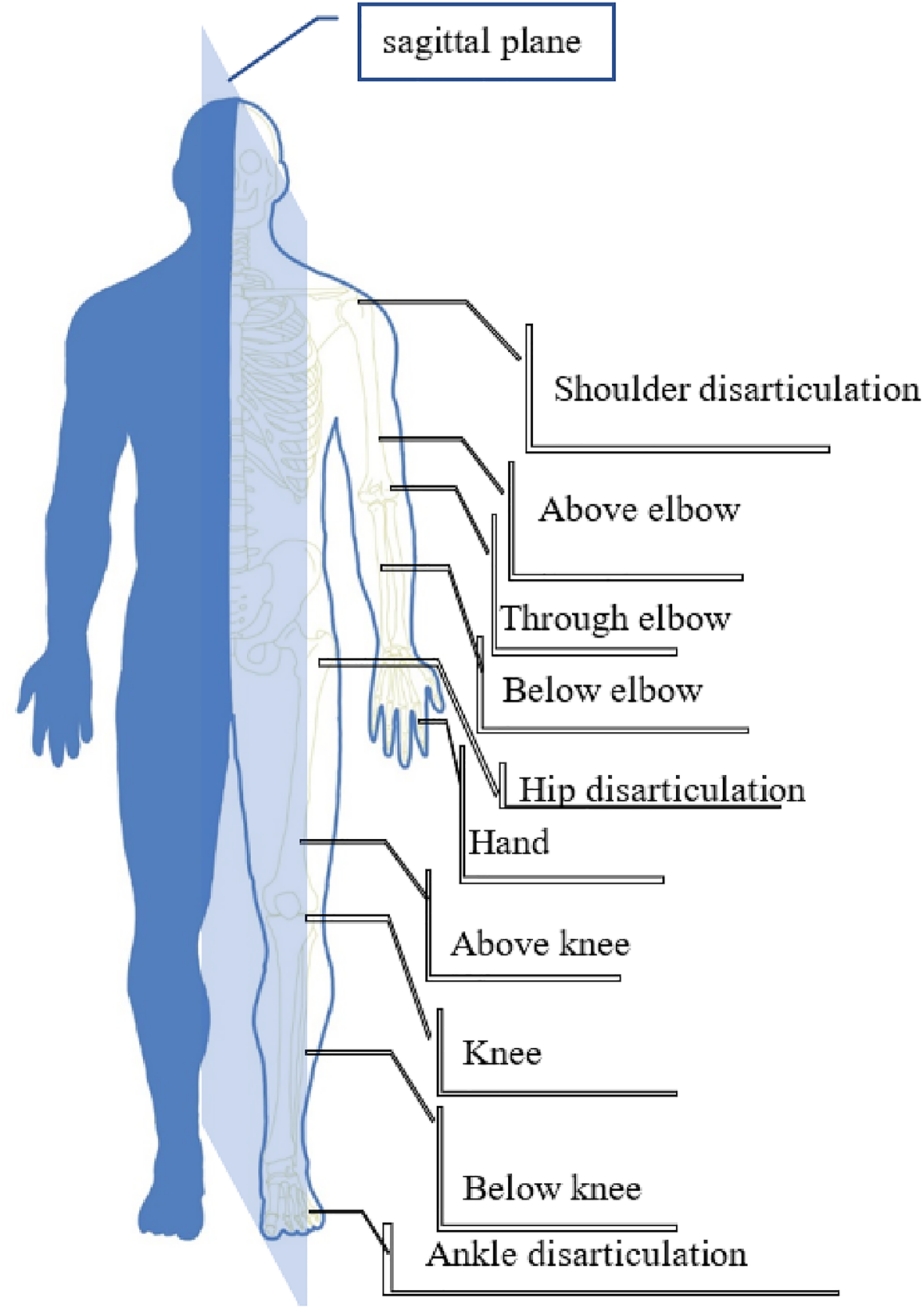
Levels of amputation in upper and lower limbs.

Globally, thousands suffer every year from complications of various diseases, such as diabetes, circulatory and vascular disease, trauma, and cancer which could lead to limb amputations^1^. Limb amputation significantly reduces the quality of life (QOL)^2^ particularly in case of lower limb amputation as it impedes amputees’ mobility^3^. Amputation levels, as shown in Figure 1, are classified into upper and lower limbs. According to^4^, lower limb amputations represent almost 97% of amputations in the United States out of 1.7 million amputees’ population. This indicates the significance of lower limb problem to be handled. On the Egyptian local level, databases about amputation levels are not comprehensively updated. However, the trend of higher lower limb extremity, represented in the United States, can be expected to take place also in Egypt. A supportive argument for this expectation is the high percentage of Diabetes in Egypt where almost 0.5% of them suffer lower limb extremity^5^. Diabetes, with its consequences of vascular diseases, results in more than 90% of the lower limb extremity^6^. Out of the Trade map website about export and import data; the documented import bill of Prosthetics and Orthotics (P&O) costs Egypt at least 600 Million L.E. in 2020 based on trade map statistics for HS-code: 9021–10,31–39 up to 1 billion L.E. according to governmental figures^7^. A greater portion of P&O import bill is not documented because of the non-counted direct purchase of the amputees abroad. This results in an untrusted database about the amputation levels and the P&O parts. The Egyptian state declared its intention in tackling this problem as represented in the conference of “Different, We Are Able’’ which is held in Cairo 2018. The state started to merge the efforts of all related local authorities and individual experts into single consortium. The activities of this consortium are putting rules for providing high-level of medical services, integrating amputees in society, establishing a comprehensive database for people with physical disabilities, and following up the process of an integrated industrial complex to localize and transfer technology for manufacturing prosthetic limbs to overcome the market gap^8^. This process requires; from another point, building well-trained manpower resources, to have an accredited professional education program and to start local Research and Development R&D capacity to sustain the localization process of P&O industry. This orientation is ensured by funding scientific and applied projects from the Egyptian state as mentioned in the acknowledgment.

In order to improve the amputee’s QOL with lower limb extremity; lower prosthetic limbs are designed to retrieve some movement functions^9^ and substitute the missing limb^10^. During rehabilitation, temporary lower limb prosthetics are used to make amputees accustomed to walking and performing daily life activities safely. The prosthetics are designed to satisfy the functions of the lost limb namely, shock absorption, weight-bearing stability, and progression.

More concern is given to the ankle foot and the below knee amputation due to their higher statistics^11^. It goes without saying that ankle foot manufacturing is a subset of the whole below knee set.

The required functions of the lost limb depend on the expected activities of the amputee. The prosthetic foot affects the posture, walking correctness, and the loading degree on the joints. Prosthetic foot takes different shapes depending on the severity of disability and functionality and therefore suitable designs and materials are chosen for each case^12^. These variables affect the prosthetic foot design and consequently the manufacturing technology adopted in Egypt.

In brief, the localization chances of lower limb prosthetic foot are studied in terms of the available technologies in Egypt. The factors affecting the status of the Egyptian manufacturing capabilities are discussed, namely the number of target amputees, definition of amputation levels, prosthetics’ design concepts, material selection, technology readiness level in Egypt for the different manufacturing alternatives. Consequently, a value chain, to control such an industry in the Egyptian state, is proposed.

### Amputation level of lower limb prosthesis: material and design classification

**Figure 2:**
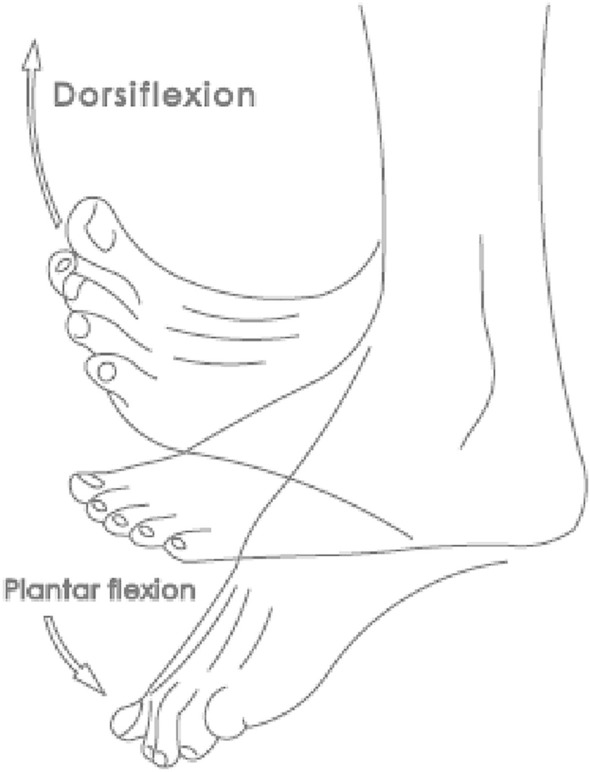
Anatomical terms of the lower limb movement.

As a normal procedure in selecting a suitable prosthetic foot design, the potential level of amputees; according to their mobility and capability to use lower limb prosthesis, is assessed by a physician then amputees are assigned a K-level which has values of K0, K1, K2, K3, or K4. This classification determines the ability of amputees to safely utilize the prosthetic foot, where K0 represents amputees who do not have the ability to walk safely without assistance while K4 represents amputees who can use prosthesis effortlessly and perform dynamic activities^13^. For low K-level amputees, solid-ankle-cushion-heel (SACH) foot is considered the most suitable option. SACH foot is the most basic type of prosthetic foot, consisting of a solid foot shaped block, usually made of wood, and combined with an aluminum pylon to join the foot to the socket. This type of prosthetic foot provides supporting function and basic mobility through a simple hinge to mimic the ankle joint motion in sagittal plane, see Fig. 1. In the late 1950s, SACH was evolved towards better simulating functions of the human foot and ankle complex. This prosthetic foot is manufactured from poplar wood keel with plywood reinforcement. Multi-axial ankle–foot mechanism was designed to accommodate uneven terrain not just plantar and dorsiflexion, as in \* MERGEFORMAT Fig. 2, in the sagittal plane. This design used a stiff anterior keel or leaf spring, made initially of Delrin and subsequently of phenolic and Fiberglas materials and high strength carbon plates, to store spring potential energy through deformation of the keel in mid to late stance and return a portion of this energy for propulsion in the absence of active ankle plantar flexors.

However, SACH foot is not suitable for higher K-level amputees as it loses a large amount of energy during gait cycle, therefore a new design was created for high K-level amputees. Energy-storage-and-return (ESR) foot is the new design which started after the launching of the Seattle Foot^14^. ESR provides mobility and convenience for users with high K-levels as it is designed with elastic materials. These materials deform under loading, then stores potential energy that is later released in the gait cycle which allows the foot to return to its original shape^15^.

Composites are used normally as materials in fabrication of ESR. These composites are reinforced with either carbon or glass fibers. Compared to other materials, composites are characterized by superior strength to weight and exceptional biocompatibility^16^. Composites can improve the gait efficiency by cumulating, storing, and then releasing energy during the gait cycle which is essential in ESR design. The efficiency primarily depends on the prosthetic foot design as well as the composite parameters such as fiber selection, fiber form, type of combination, mass content, as well as the design of the prosthesis^17^.

In brief, the design of the lower limb prosthetic has traditional and modern approaches. The traditional one goes back to 1980, where the foot types, as mentioned earlier, are classified upon the number of axes namely, single axis SACH, multi-axis, and dynamic response. This traditional classification comprises what is called conventional foot (CF) types. The modern classification is based on the energy timeline which divide the prosthetic foot into CF, ESR, and bionic foot^18^. Despite the ESR prosthetic foot being able to store and release mechanical energy, there is no net positive output work to help the amputee in forward progression. This ESR prosthetic foot does not have the ability to adapt to different terrain. The amputees with passive foot prostheses suffer and face difficulties during walking on slopes^19,20^. Also, the smooth roll-over shape of the human ankle–foot which was presented by Hansen^21^ affects the walking efficiency and performance. Hence, the prosthetic foot should have roll-over characteristics similar to human. Hansen also^22^ developed the third type of feet in the modern classification which is the bionic foot. It is the ankle–foot prosthesis, which is capable of automatically adapting to different walking surfaces and changing the ankle joint impedance from low to high throughout stance phase. The main problem of adding actuators to the ankle–foot prosthesis is the increasing of the total prosthetic weight which affects the amputee comfort.

The following paragraph will discuss which type of feet is more appealing to the planned industrial complex regarding the expected demand statistics, its economic burden and needed technology.

### Technology readiness level for prosthetic manufacturing

Technology readiness level (TRL) is an agreed-upon method to assess the maturity of certain technology. It is a nine-level system as shown in Table 1. Level 1 is just observation of basic principles. Then TRL develops across different levels of the concept formulation, proof, validation till level 9 of practical proof in an operational environment^23^.

**Table 1:** Levels of TRL.

1	Observation of basic principles
2	Concept formulation of Technology
3	Experimental proof of concept
4	Technology validation in lab
5	Technology validation in relevant environment
6	Technology demonstration in relevant environment
7	System modeling or prototype demonstration in operational environment
8	System completion and qualification
9	Actual system proof in an operational environment

As mentioned previously, the aim of this work is to evaluate the current technologies available in Egypt to start localization of manufacturing the lower limb. This evaluation depends accordingly on the TRL of each type of the prosthetic feet prosthesis illustrated in Table 2. Based on previous literature^12,14,16,24,25,26^, Table 2 presents the four types of feet according to the timeline accompanied with their descriptions in terms of their advantages and disadvantages. The TRL of each type is estimated regarding the manufacturing capabilities in the Egyptian market as shown in \* MERGEFORMAT Table 3. TRL of the first type is 7 and the technology required for the CF foot is considered "not advanced". However, the second type of the prosthetic feet is selected for localization in Egypt due to the three following reasons:

- The simple non-advanced technology in the low-income countries is the governing reason for selecting CF foot due to its relatively low price and simple maintenance. However, CF foot meets the needs of utmost K2 amputees. Getting the amputee feels more natural walking pattern (gait) requires mimicking the dynamics of an anatomical foot. The second type of feet in Table 2, ESR foot, fulfills partially these dynamics. But cost wise, it is expensive and costs more than 5 thousand USD depending on the material and the design such as multi-axial and microprocessor. Even the bionic foot in Table 2 may cost more than 100 thousand USD in western countries^27,28^. In other words, keeping a low level of mimicking the natural foot meets the cost limitation of the low-income country amputee. However, it reduces the QOL and negatively affects the surrounding relatives and the whole community’s efficiency. Therefore, selecting a relatively higher degree technology would influence positively the QOL of the amputees and their relatives.
- The import bill of the prosthetic foot to Egypt is mainly attributed to the higher added value of advanced technology foot. This is attributed to the presence of manufacturing centers for CF foot in Egypt. Also, this is evidenced by the market report about growing market in the Middle East especially for advanced prosthetic feet^29^.
- TRL of ESR foot is promising. Except for the carbon fiber part, the manufacturing technologies of the foot components are available and mature in Egypt. The carbon foot part itself is processed manually. The chain of the processes comprises of fabric cutting, orientation, stacking, resin infusion, curing, trimming, and machining. The manufacturing process of ESR using composite material has gone through different phases. Starting from manual hand layup which is considered the simplest technique to produce layers of laminates in the composite as it is a low-cost tool and uses room temperature-cured resins. However, this technique is time-consuming and the composite is prone to air bubble formation^30^. The opportunity to automate part of this chain is highly potential by some technologies not available in Egypt like resin transfer molding RTM or Resin pre-impregnated Fabric PREPREG. RTM is more potential to be realized as it is not complicated technology involves the pumping of resin into a closed die filled with stacked carbon fiber fabric in the required foot preform as a one part or more according to the design, see \* MERGEFORMAT Fig. 3. Vacuum Assisted Resin Transfer Molding (VARTM) is another form of RTM technology. VARTM technology is used for production on a small scale, therefore it is utilized for producing prototypes^31^. The most advanced techniques are pressurized Resin Transfer Molding (RTM)^32^ and the use of pre-impregnated carbon fabrics with resin (PREPREG)^33^ to ensure good quality.

**Table 2:** Technologies of manufacturing lower limb prosthesis.

#	Prosthetic Foot Technology	Description	Advantage	Disadvantage
1	conventional foot (CF)^34^	SACH (Solid Ankle Cushioned Heel) is non-articulated foot which has no moving parts and manufactured from poplar wood keel with plywood reinforcement	1. Simple Design 2. Low cost	1. Rigid keel that cannot bend 2. Poor Toe off 3. Fixed Heel height 4. Usually limited to K1 and K2
2	Mechanical energy storing and returning (ESR)^35^	ESR constructed of carbon fiber or fiber glass composite which works as leaf spring that allows mechanical energy storage during stance and releasing it during push-off to support swing phase	1. able to store and release mechanical energy 2. Possible Push off gait is split-toe feature allows to mimic inversion and eversion 3. Decrease the impact and stress on the sound leg during gait 4. Not limited to K2	1. High cost 2. does not have the ability to adapt to different terrain
3	Micro-processor Foot^36^	Prosthetic ankle–foot which has motors, sensors, and ESR. It has also real time microprocessor control used to adjust and control the damping and braking resistance during daily life activities	1. Able to respond to various terrains and environment 2. Easy ankle adjustments and alignment to different terrains and slopes 3. Improve amputee balance and mobility	1. Very Expensive 2. Cannot provide positive energy to the prosthetic foot 3. Need to be charged
4	Bionic foot^37^	It is powered controlled prosthetic ankle–foot which can provide positive power to drive ankle–foot movement. It has high power motor to support push-off and dorsiflexion	1. Provide positive power 2. Reduce metabolic energy computation in transtibial amputees	1. Very expensive 2. Heavy weight due to motor weight and battery

**Table 3:** Components breakdown of the manufacturing technologies for lower limb prosthesis.

#	Prosthetic Foot Technology	Sketch	Component/ Manufacturing capability	Estimated TRL
1	conventional foot (CF)		1- Rigid wooden keel^38^ (CNC) or Polypropylene PP 2- Rubber shell or Polyurethane foam (Manual molding)^39^ 3- Cushion of PP or polyoxymethylene POM^39^ (Molding)	7
2	Mechanical energy storing and returning (ESR)		1- Adaptor (CNC machining) 2 & 3- Polymer composite system (Hand layup, Vacuum Infusion, Resin transfer molding RTM, Prepreg molding)^40^ 4- Joining by metallic bolts and adhesives	3
3	Micro-processor Foot		1- Adaptor (CNC machining) 2- Microprocessor to control the ankle motion motor to reduce risk during walking on slopes and everyday terrain^41^ 3- ESR foot as in number 2	2
4	Bionic foot	It is powered controlled prosthetic ankle–foot which can provide positive power to drive ankle–foot movement. It has high power motor to support push-off and dorsiflexion	1. Provide positive power 2. Reduce metabolic energy computation in transtibial amputees^42^	1

**Figure 3:**
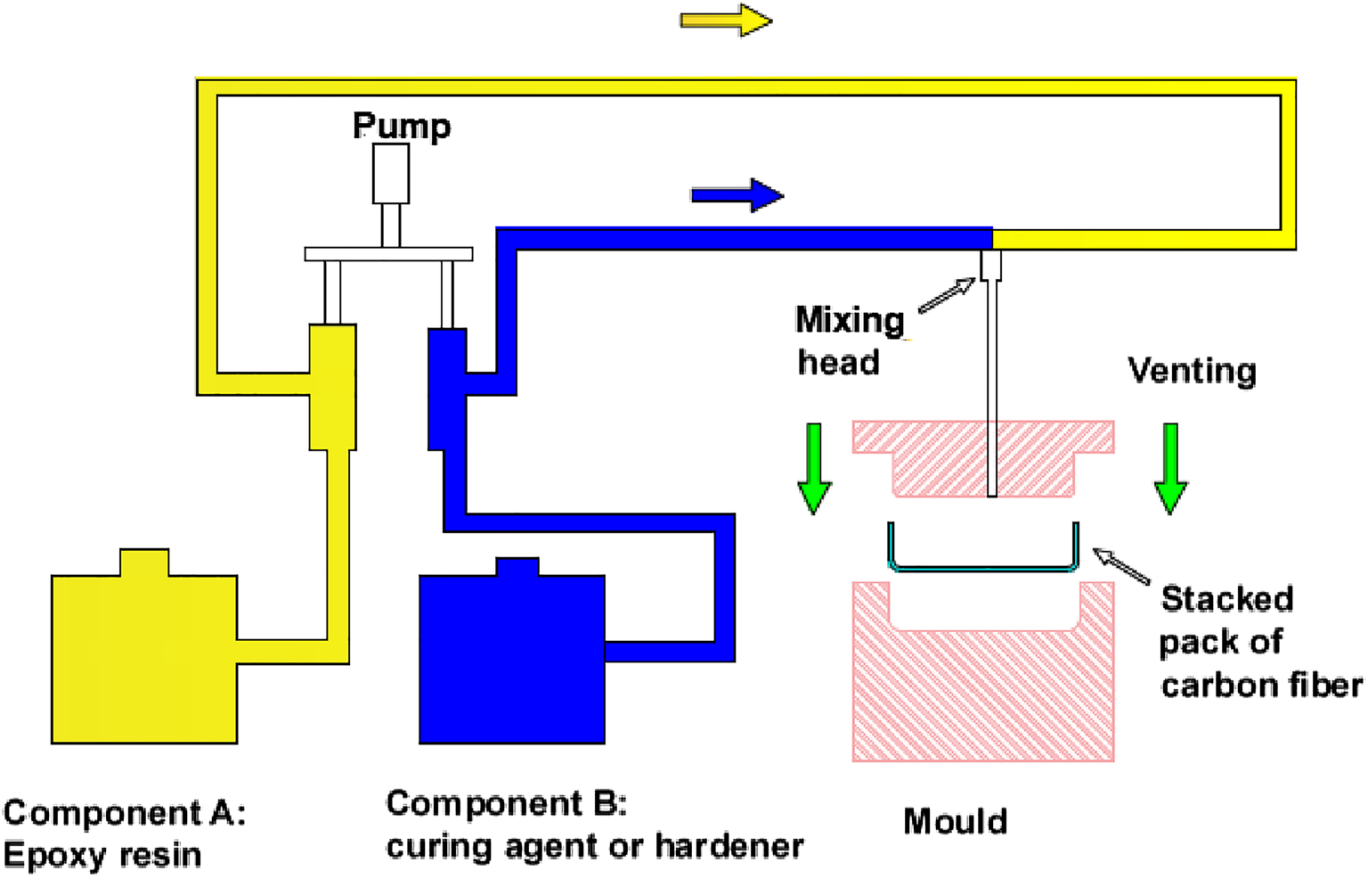
Schematic of RTM Principle.

### Methodology of prosthetic ESR foot manufacturing

**Figure 4:**
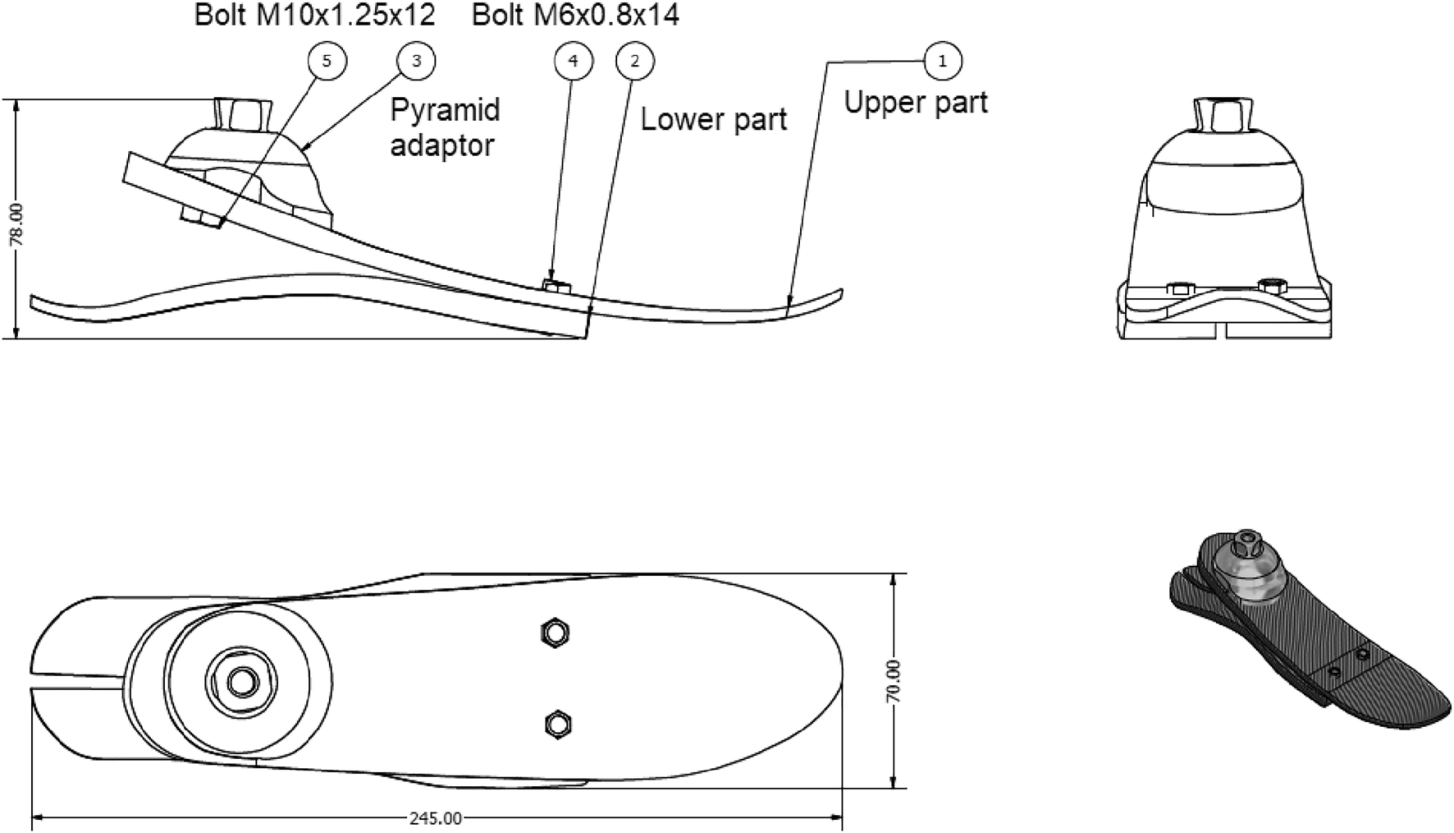
Selected common size of prosthesis foot.

Following a modular design would help in the industrialization of the P&O parts and in the definition of the required TRL that should be met. The prothesis consists of several components^1^. Figure 4 shows the selected common size of 27 under investigation. The components of the foot can be classified as modular parts. The modular components are like the pyramid metallic adaptor and the bolts. The upper and lower parts of the foot itself are also considered modular products as they can be classified to specific modular sizes. Non modular part is like the socket which connects the pyramid with the amputee remaining part of the leg.

The proposed methodology of localizing the prosthetic foot manufacturing in Egypt is as follows:

- Designing of the prosthetic foot where the foot breakdown consists of modular parts to help mass production, maintenance and interchangeability. The proposed design is checked by modeling regarding the foot endurance to the expected stresses;
- Selection of the manufacturing method with respect to the TRL in Egypt and the product complexity;
- Testing of the ESR foot;
- Developing a value chain regarding the results and discussion.

## Results and discussion

### a. Modeling of prosthetic ESR foot

**Figure 5:**
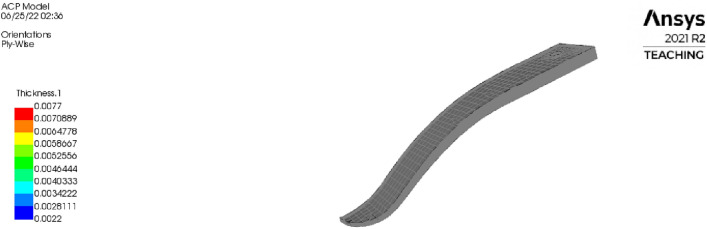
Solid lower part of the ESR foot obtained through ANSYS ACP module.

To model static tests on the prosthetic foot, ANSYS workbench was used. ACP module was used to develop solid composite parts, the mechanical model module to develop a meshed platform for the static test, and finally static structural module to perform the static test using the parts developed in the other two modules. In the ACP module, the materials used for the parts are wet epoxy carbon woven and resin epoxy as built-in materials in the ANSYS engineering data sources. With the aid of the stack-up fabric layers from the manufacturing stage, a solid model was created using the oriented selection set and resin epoxy as its global drop-off material as shown in Fig. 5.

**Figure 6:**
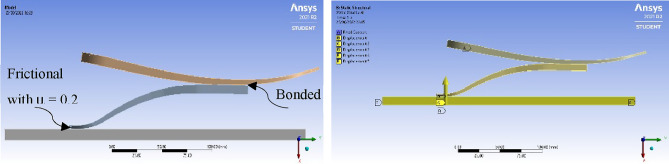
Contacts and boundary conditions used in ANSYS Static Structural module, respectively.

**Figure 7:**
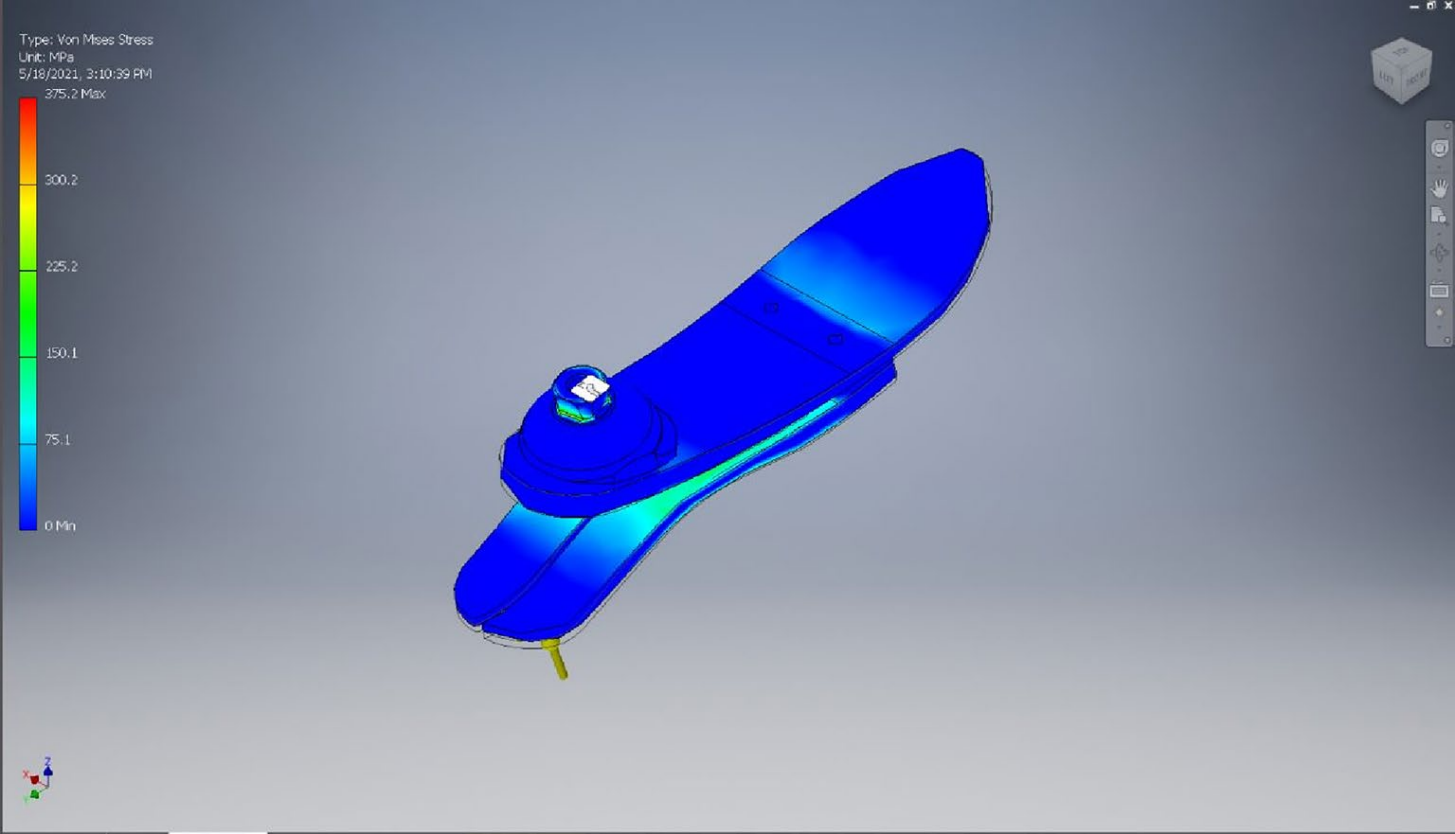
Von Mises Stress distribution of the foot.

Parts created in the previous modules were transferred as solid bodies to the model section in this module. Since the parts were not pre-assembled on the CAD software, the parts were assembled in ANSYS Mechanical. Contacts and boundary conditions were applied to the parts to perform the static tests. For the contact between the lower parts and the upper part was estimated as a bonded connection. For the contact between the lower parts and the platform, it was estimated as a frictional surface with a coefficient of friction equal to 0.2. For the formulations, the frictional contact was formulated based on Augmented Lagrange while the bonded contact was formulated automatically by ANSYS. To mimic the conditions of the ISO static test, multiple constraints were applied to ensure the same behavior throughout the simulation. Initially, the holes for the pyramid were defined as fixed supports. Then, the platform was given a displacement of 20 mm in the vertical direction while keeping the side face from moving in the horizontal and transverse direction as shown in Fig. 6. Finally, Fig. 7 shows the checking process regarding the maximum allowable von mises stress.

### b. Manufacturing of prosthetic ESR foot

**Figure 8:**
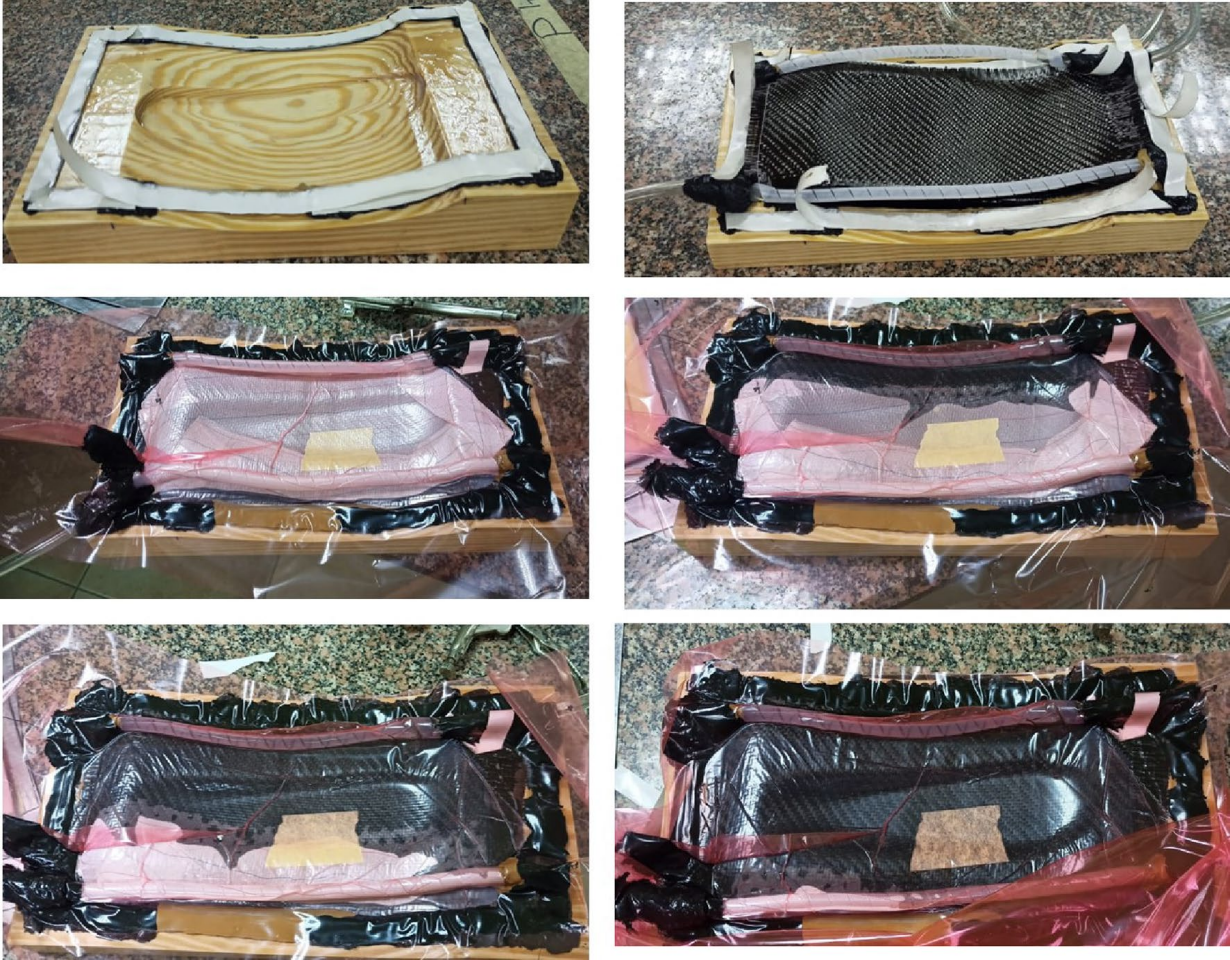
Heel manufacturing by vacuum infusion.

**Figure 9:**
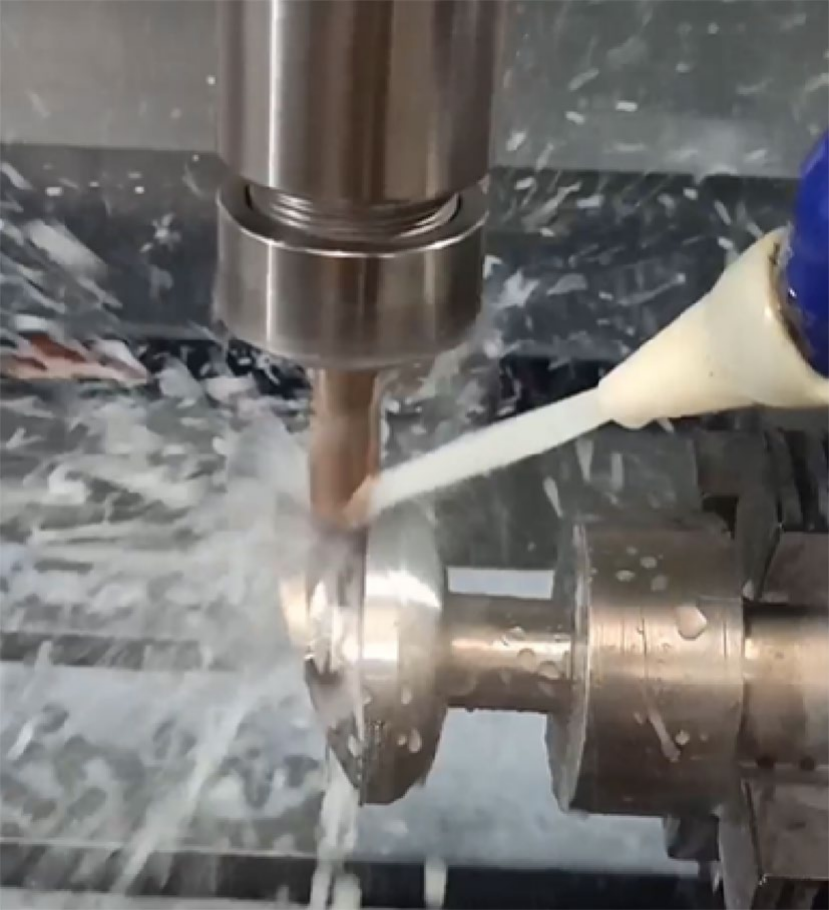
Adapter manufacturing using CNC.

Manufacturing of the prosthetic foot parts is carried out briefly as follows. Carbon fiber fabrics are used in manufacturing the upper- and lower-foot parts. They are of density of 200 g/m^2^, Modulus of elasticity 200–588 GPa and tensile strength of 2800–5490 MPa. Upper and lower carbon fiber parts are prototyped using assisted vacuum hand layup as shown in Fig. 8. Then they are at room temperature cured overnight. Accurate dimensions of the foot parts are reached by further machining. Metallic adapter between the composite and the knee is locally machined by 3 axis computerized numerical machine as shown in Fig. 9. Bolts and adhesives are modular market items.

### c. Testing of the prosthetic ESR foot

**Figure 10:**
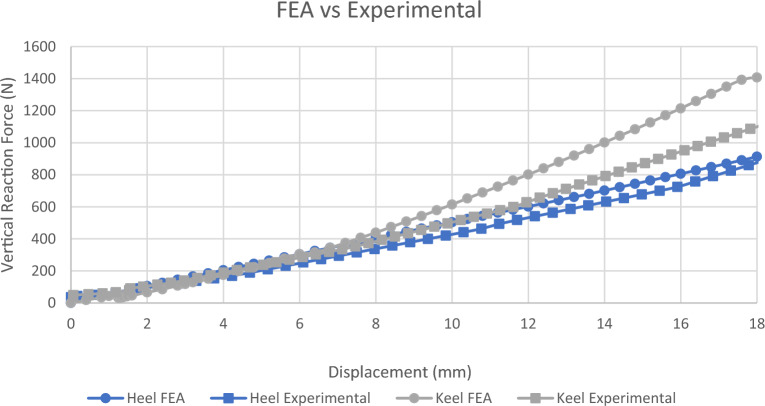
Comparison between simulation and experimental proof static tests.

After foot manufacturing, static compression tests are carried out to check foot endurance and to validate modeling. By comparing the results obtained from testing the manufactured model using FEA and experimental testing as shown in Fig. 10, FEA testing shows higher load values due to the usage of bonded contact between the upper and the lower parts of the ESR foot which constrains the movement of the body leading to a higher recorded load than the actual load formed in the ESR foot when tested experimentally. Thus, the acceptance of the FEA model using safety factor measurements will automatically mean the acceptance of the manufactured model.

**Figure 11:**
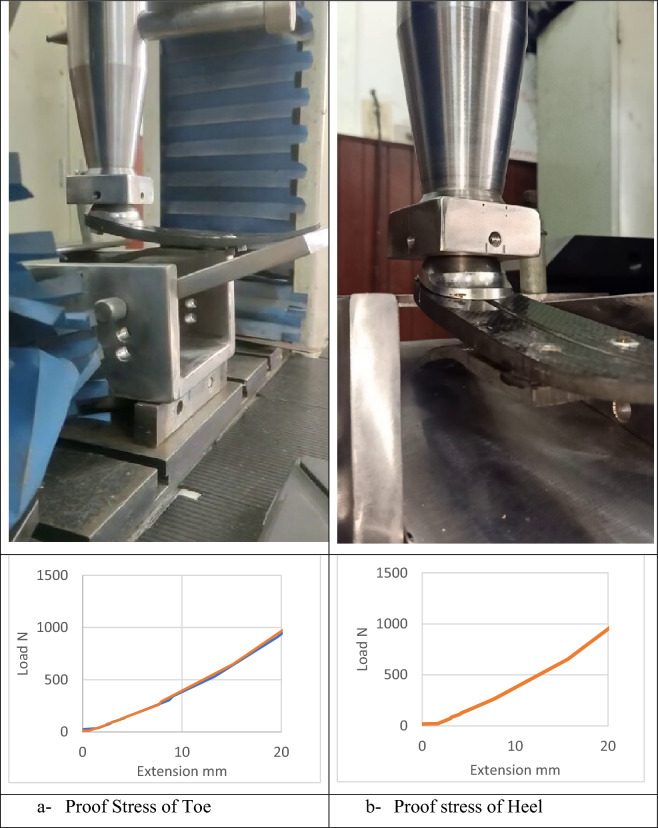

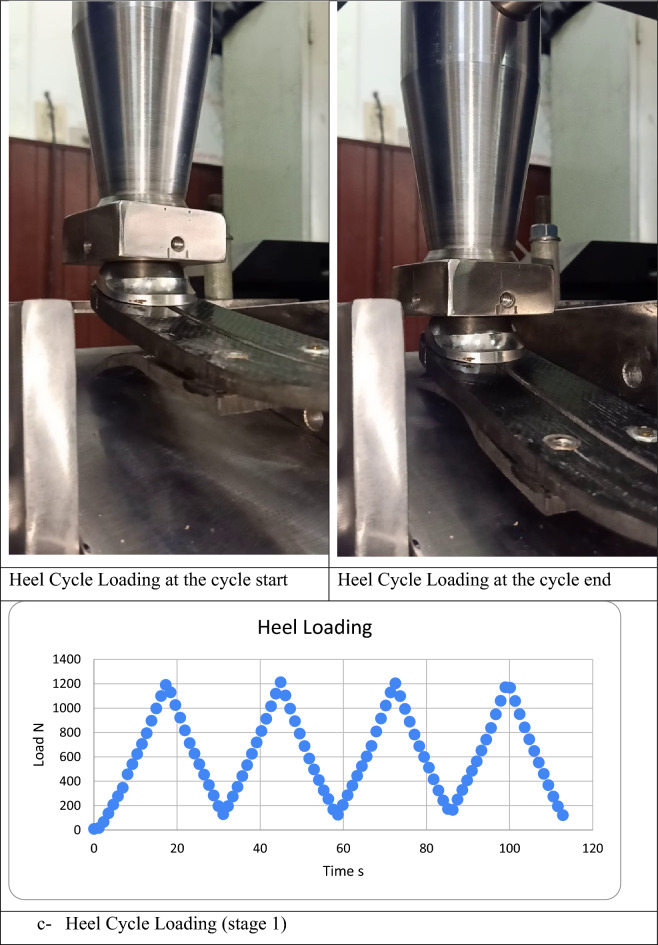
Mechanical testing of the foot assembly.

The entire assembly of the prosthetic foot components took place after fabrication, and it went through mechanical testing following ISO22675 guidelines, as detailed in Table 4. Figure 11 illustrates some of the mechanical tests that were applied to the assembled foot. Following the completion of these tests, a thorough inspection was conducted to identify any visual defects, such as delamination, dismounting, or indentation marks resulting from over-tightening. The mechanical tests described in this study adhered to ISO22675 standards and did not involve any experiments on amputees or human subjects.

**Table 4:** ISO22675 Testing Procedures^43^.

Test	Procedures	Notes	Acceptance Criteria
Static proof	In heel loading, apply the test force F1 at angle γ1. While loading, apply the test force F2 at angle γ2. Increase the force at a rate of between 100 N/s and 250 N/s to the proof test force F1sp of the relevant test loading level. (Maintain the F1sp and F2sp force for 30 ± 3 s) then decrease the test load to zero	Obtain γ1, γ2, F1, and F2 from tables 8 and 9 in ISO	Check Fig. 9 in ISO
Static ultimate	Apply F1 to the heel and increase it at a rate between 100 N/s and 250 N/s until the test sample fails, or the test force, F1 attains the value of the ultimate test force F1su, upper level of the relevant test loading level, specified in without failure of the test sample. The procedure is repeatedly done for the keel at and F2 and γ2	Obtain γ1, γ2, F1, and F2 from tables 8 and 9 in ISO	Check Fig. 10 in ISO
Cyclic	Stage (1): Successively applying static heel then load test at a max test force of about F_cmax_	Obtain γ1, γ2, γ_fcmin_, F_cmin_, F1_cmax_, and F_cmax_ from tables 8 and 9 in ISO	End test without failure
	Stage (2): Pulsating test F_c_(t) or F_c_(γ) with variable γ(t) at a frequency of 0.5 Hz to 3 Hz		
Final static proof	Same Procedures as the static proofing test	–	–

We confirm that all the tests in this work were not conducted on human subjects. Additionally, all methods were performed in accordance with the relevant guidelines and regulations.

### d. Value chain for the manufacturing of the Prosthetic ESR Foot

The value chain for the prosthetic foot in Egypt would be better to be designed and oriented not to be left for market driving forces. This objective is in agreement with the governmental policy. To build this value chain, the sub-objectives are targeted as following:

e. Well definition of the number of needed production lines to provide service. This will be based on the geographical distribution of amputees and the available trained/ educated manpower;
f. Standardizing the health and insurance services for the people with disabilities in a proper management procedure;
g. Best value-to-money or highest feasible QOL with affordable price.

**Figure 12:**
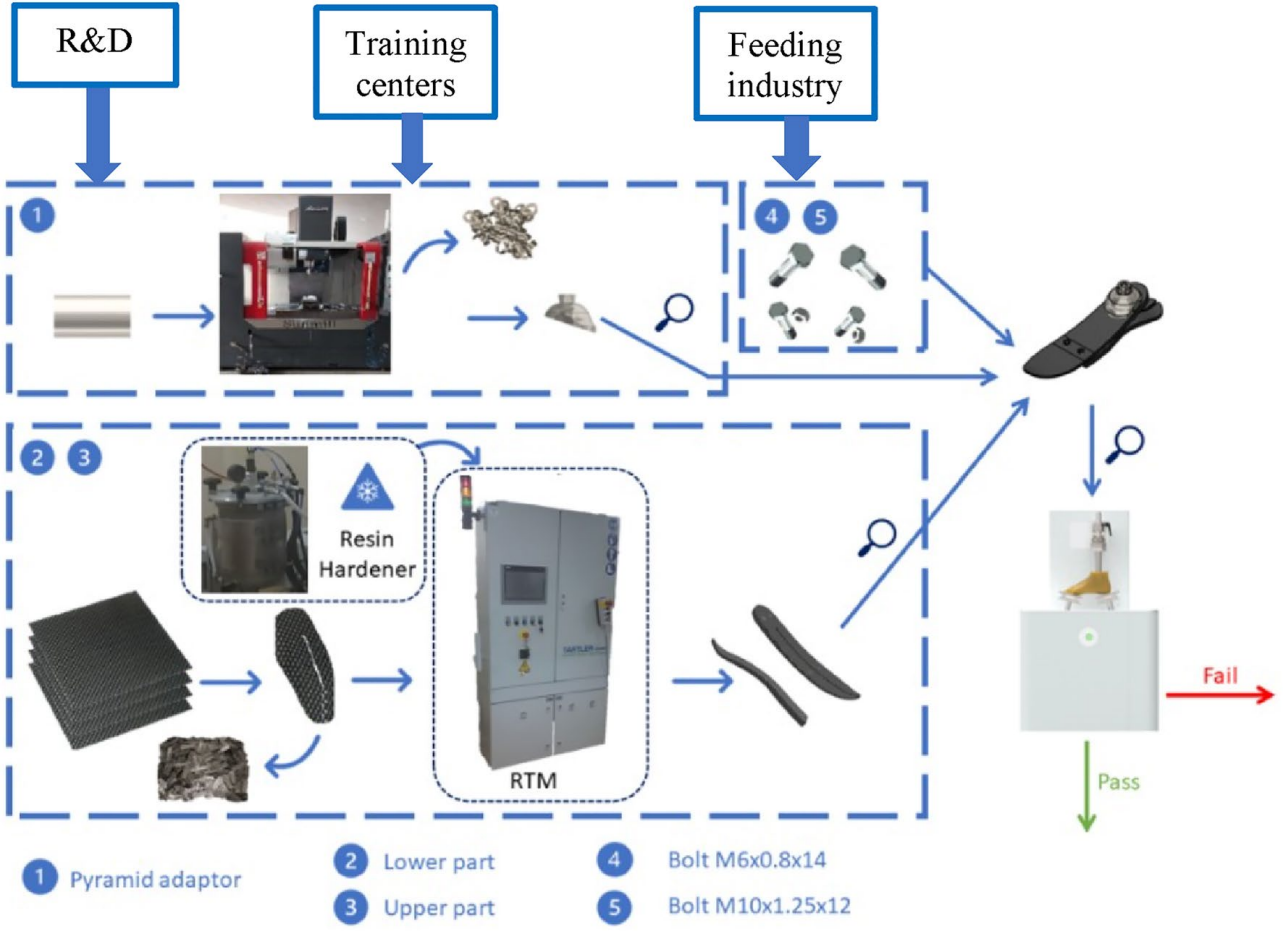
Manufacturing value chain of ESR foot manufacturing.

Regarding the third point mentioned above and based on the TRL study; the supposed manufacturing line for the prosthetic foot is shown in Fig. 12. There are other activity centers which would definitely serve the manufacturing value chain as shown in the three text boxes regarding research and development R&D, training and educational centers and other feeding industries. These activities will promote other actions. For instance, the presence of national non-biased labs in compliance with ISO17025 to carry on the required tests like ISO22675 issued by the technical committee TC168. On the training and educational level, the certification of manpower will be necessary done according to accreditation criteria set by entities like International Society for Prosthetics and Orthotics ISPO. Feeding industries will also be constrained by the biomedical material requirements. On the other hand, the products will be evaluated, and the product design will be updated accordingly.

This value chain is still a global one as it depends on imported materials like carbon fiber. But the use of other alternative local materials like natural fiber will make the chain more self-sufficient. This suggested value chain focuses on the prosthetic foot composite part only but the other components in touch with the patient like the socket are not considered in this work. This leaves a chance for the current practices and the available prosthetists to adapt gradually with the new technologies as recommended in^44^.

## Conclusion

Know-how transfer and localization of ESR Prosthetic Feet Manufacturing Processes in Egypt, as shown throughout this work, are essential to cover the community needs and comply with the state vision. The TRL of the product components shows promise for proceeding with the establishment and growth of this value chain.

The study proves the possibility of prosthetic feet manufacturing using available material and manufacturing technologies. Available materials include carbon fiber fabrics, binding epoxy materials, metallic adapters and joints. Available technologies like VARTM technology for resin impregnation in fiber fabrics and conventional machining for metallic parts.

Also testing of the prosthetic foot is an important part of the value chain. Testing is carried out according to ISO 22,675 to localize the product accreditation in the future.

In the near future, prosthetic feet manufacturing will open the door to foster the use of many non-localized technologies in resin impregnation for engineering products of fabric composites. The impact of localizing these new technologies of resin impregnation is not limited to the medical rehabilitation, but it will disseminate to other versatile aspects such as automobile industry^45^. It is worthy noted that there are other prospective technologies for prosthetic feet manufacturing like 3d printing which can be followed.

## Data Availability

All data generated or analyzed during this study are included in this published article.
